# Postharvest Management of Fruits: Navigating the Affordability, Efficacy, and Non-Destructive Quality Evaluation of Thermal Versus Non-Thermal Interventions

**DOI:** 10.3390/foods15183349

**Published:** 2026-09-21

**Authors:** Kishenthi Kerisnan, Kivaandra Dayaa Rao Ramarao, Sze-Looi Song, Chandran Somasundram, Zuliana Razali, Sarmila Muthukrishan, Saiful Irwan Zubairi, Mohammed Wasim Siddiqui

**Affiliations:** 1 Institute for Advanced Studies, Universiti Malaya, Kuala Lumpur 50603, Malaysia; 25079946@siswa.um.edu.my; 2 Institute for Biological Sciences, Faculty of Science, Universiti Malaya, Kuala Lumpur 50603, Malaysia; zuliana@um.edu.my; 3 Department of Science and Technology Studies, Faculty of Science, Universiti Malaya, Kuala Lumpur 50603, Malaysia; sarmila_tm@um.edu.my; 4 Department of Food Sciences, Faculty of Science & Technology, Universiti Kebangsaan Malaysia, Bangi 43600, Malaysia; saiful-z@ukm.edu.my; 5 Indian Institute of Packaging, Plot E-2, MIDC Area, Cross Road B, Road No. 8, Andheri East, Mumbai 400093, India; wasim@iip-in.com

**Keywords:** fruit quality, postharvest preservation, thermal preservation technologies, non-thermal preservation technologies, economic affordability

## Abstract

Postharvest losses (PHLs) present a critical bottleneck to global food security, with fresh fruit losses reaching 23–40% due to rapid physiological deterioration, mechanical stress, and microbial decay. This review evaluates the metabolic cascades, including respiration kinetics, transpiration, and hormone-mediated factors, that govern fruit degradation. We reviewed thermal and non-thermal treatments for affordability, including thermal interventions such as hot water treatment (HWT), microwave treatment, and radio frequency treatment, alongside emerging non-thermal preservation modalities such as edible coatings, cold plasma atmospheric treatments, and ultrasound processing. The review places particular emphasis on climacteric tropical fruits such as mango, papaya, banana, and citrus, for which postharvest losses and preservation challenges are especially acute due to their climacteric nature. To address the quality tracking highlighted by modern commercial chains, this review integrates an assessment of non-destructive quality evaluation technologies, focusing on near-infrared (NIR) spectroscopy, hyperspectral imaging (HSI), and electronic noses (E-noses). Overall, this review seeks to deliver a unified blueprint, arguing that sustainable postharvest preservation can benefit from context-specific hurdle-technology concepts that align accessible postharvest treatments with monitoring networks. We synthesise current evidence on individual thermal, non-thermal, and sensing technologies and use this to motivate potential hurdle strategies and treatment–monitoring pairings for diverse fruit supply chains.

## 1. Introduction

According to the United Nations, the global population is projected to reach approximately 9.7 billion by 2050, placing unprecedented strain on natural resources and food supply chains [1]. This rapid population growth, combined with climate change risk, threatens the global food and nutritional security systems. Despite improvements in agricultural productivity in recent decades [2], geographical boundaries, increasing land degradation, and diminishing freshwater availability have reduced the capacity of primary food production as a sustainable strategy for maintaining human nutrition for global demands. Therefore, the global food system requires a fundamental transition towards optimising resource utilisation and systematically reducing inefficiencies across supply chains, particularly through minimising postharvest losses.

Postharvest management is one of the most effective intervention points for enhancing food availability, since PHLs are estimated to account for up to half of harvested produce between harvest and final consumption. These losses have significant economic, environmental, and nutritional challenges, including compromised food availability and quality, the depletion of valuable resources, and diminished nutritional value [3,4]. More than a decade ago, the Food and Agriculture Organisation of the United Nations (FAO) reported that, globally, 413 million tonnes (MT) of food were lost during agricultural production, 293 MT during postharvest handling and storage, 148 MT during processing, and 161 MT during distribution, while 280 MT were wasted at the consumption stage [5]. Worryingly, the FAO [6] recently reported that there is no discernible progress in reducing food losses since monitoring began in 2015. The proportion of food lost throughout the handling, storage, transportation, processing, and wholesale stages increased from 13.0% in 2015 to 13.3% in 2023. Fruits and vegetables accounted for the highest losses because of their high perishability and susceptibility to physiological deterioration. These losses also represent a substantial waste of water and energy and contribute to carbon emissions [7].

Among horticultural commodities, fruits are most susceptible to postharvest loss (PHL). Fruits are an important source of nutrients such as vitamins, minerals, and dietary fibres, which help in maintaining good health and minimising risks of chronic disease [8]. Global fruit production has experienced significant growth, reaching 933 MT in 2022. This represents a 63% increase compared to two decades ago, and the market itself is projected to grow to USD 870.86 billion by 2034 [9]. The growing consumer demand for fresh fruits further underscores the importance of effective postharvest management. Meeting this demand is increasingly challenging under conventional production systems because of climate variability, soil degradation, and constraints on water and other agricultural resources [10]. Reducing postharvest losses is therefore important not only for maintaining fruit quality but also for improving the efficiency with which existing fruit production contributes to food and nutritional security. However, they have a highly perishable nature, particularly in tropical and subtropical regions where cold-chain infrastructure remains limited [11,12]. Climacteric fruits such as banana, mango, and papaya are among the commodities most susceptible to postharvest deterioration because they continue to undergo active physiological and biochemical processes even after detachment from the parent plant [13]. These processes include respiration, ethylene-mediated ripening, metabolism, and transpiration, which, together with postharvest handling and storage conditions, strongly influence fruit quality and shelf life [14]. As a result, senescence is accelerated, and the fruits become more vulnerable to microbial contamination and deterioration during storage, transportation, and marketing [15]. Importantly, PHL is not just about reduction in quantity, but also focuses on deterioration in quality and nutritional value, which may occur before visible signs of spoilage become obvious. Essential bioactive compounds like ascorbic acid, carotenoids, and polyphenols often degrade considerably before visible symptoms of fungal decay or defects manifest on the fruit surface, making these losses difficult to identify via conventional visual inspection and grading methods [16].

This limitation highlights the need for both effective preservation strategies and rapid, reliable, and non-destructive assessment techniques capable of continuously monitoring internal fruit quality throughout the postharvest supply chain. Recent reviews highlight that postharvest management has relied mostly on conventional preservation approaches such as refrigeration, chemical fungicides, wax coatings, and modified storage conditions to slow down fruit deterioration and maintain market quality [17]. Although these approaches can substantially reduce postharvest losses, their long-term application is often constrained by chemical residue concerns, environmental sustainability, and growing customer preference for safer and chemical-free foods [17]. Consequently, extensive research has focused on thermal and non-thermal preservation technologies that have been shown to extend fruit shelf life while preserving physicochemical, nutritional, and sensory quality [18,19,20]. In parallel, technological advances have enabled the integration of smart sensing systems, automation, and data-driven decision-making throughout the postharvest supply chain. Emerging postharvest preservation technologies, such as thermal- and non-thermal-based methods, have gained considerable attention for their ability to maintain the nutritional quality of fresh produce. However, their efficacy, equipment cost, energy consumption, operational complexity, and translation into industrial-scale applications vary considerably. Selecting an appropriate postharvest intervention therefore requires careful consideration of its biological efficacy, affordability, practical implementation, scalability, and compatibility with existing supply chain infrastructure.

Despite significant advances in postharvest preservation technologies, most current reviews, including recent overviews of emerging postharvest technologies and sustainable postharvest innovations, have primarily focused on the biological and technological performance outcomes, such as antioxidant retention, microbial inhibition, maintenance of physiological quality, and extension of shelf life [21]. However, the practical potential of these technologies depends on a broader set of considerations, including affordability, infrastructure requirements, operational complexity, scalability, and compatibility with different production systems. These practical implementation factors are rarely evaluated collectively, making it difficult for researchers, industry practitioners, and policymakers to evaluate the suitability of different technologies under diverse contexts. Furthermore, advances in non-destructive quality assessment technologies, including near-infrared (NIR) spectroscopy, hyperspectral imaging (HSI), and electronic noses (E-noses), are often reviewed independently of preservation strategies, despite their complementary role in monitoring internal fruit quality throughout postharvest handling. This limits a comprehensive understanding of how preservation efficacy can be balanced with effective monitoring and practical implementation. Consequently, the existing literature offers limited insights into the delicate balance between preservation efficacy and continuous quality monitoring, coupled with practical implementation potential.

In contrast to previous overviews that primarily synthesise biological outcomes and describe individual technologies in isolation [21,22], this review develops a decision-oriented framework that jointly evaluates preservation and monitoring strategies across five implementation dimensions. We synthesise current evidence on individual thermal and non-thermal preservation technologies and critically compare their preservation efficacy, economic affordability, operational complexity, commercial scalability, and suitability across different production systems. This review focuses primarily on the postharvest preservation of tropical fruits, with particular emphasis on highly climacteric commodities such as banana, mango, and papaya. These fruits experience disproportionately high postharvest losses due to their rapid ripening and sensitivity as climacteric fruits. Occasional examples from other fruit groups are also cited to illustrate specific technological principles or operational constraints. Furthermore, conventional storage technologies such as refrigeration, freezing, controlled-atmosphere (CA) storage, and modified-atmosphere packaging (MAP) are recognised as fundamental postharvest preservation approaches and are discussed where relevant throughout the manuscript. However, they are not included as individual technologies within the comparative evaluation framework because the present review specifically focuses on intervention-based preservation strategies rather than continuous storage systems [21,23]. We further integrate emerging non-destructive quality-assessment technologies, including NIR spectroscopy, HSI, and E-noses, with preservation strategies to consider their complementary roles in postharvest management. Collectively, this framework provides researchers, industry stakeholders, and policymakers with a more practical foundation to ease the selection of context-appropriate postharvest strategies that balance technical performance, economic feasibility, and sustainable implementation.

## 2. Review Methodology

This narrative review synthesised relevant peer-reviewed articles and review papers written in English on postharvest preservation and non-destructive quality monitoring of fresh fruits. Relevant literature was identified from Web of Science, Scopus, ScienceDirect, and Google Scholar databases using the following keywords: “postharvest”, “fruit”, “thermal treatment”, “non-thermal treatment”, “edible coating”, “cold plasma”, “ultrasound”, “microwave”, “radiofrequency”, “non-destructive”, “near-infrared spectroscopy”, “hyperspectral imaging”, and “electronic nose”. The primary search spanned the years 2000 to 2024, with earlier studies included only if they were technically foundational. Where necessary, websites such as government portals were accessed to retrieve relevant data. We focused on studies that (i) investigated postharvest preservation or quality monitoring of fresh fruits, (ii) reported quantitative outcomes such as changes in shelf life, decay incidence, physicochemical attributes, or quality-prediction accuracy, and (iii) described the intervention or sensing conditions in sufficient detail. Exclusion criteria included studies on minimally processed products, non-fruit type postharvest produce, and papers that reported purely mechanistic modelling without experimental validation.

## 3. Current Postharvest Losses and Regional Challenges

Postharvest losses, in particular for fruit commodities, remain a major global challenge in which an estimated 23–40% of fruit production is lost annually due to biological deterioration and inefficiencies throughout the supply chain. The magnitude and causes of these losses vary considerably among regions owing to differences in climate, infrastructure, transportation systems, market integration, and access to postharvest technologies [24]. Following harvest, fruits, especially climacteric fruits, remain metabolically active and continue to undergo respiration, transpiration, ripening, and senescence in the absence of physiological support from the parent plant [15]. These intrinsic physiological processes are further aggravated by external factors such as mechanical injury, inadequate temperature management, poor packaging, and limited cold-chain facilities, which accelerate quality deterioration and increase susceptibility to microbial infection [25,26,27].

Regional disparities strongly influence the extent of postharvest losses. Developing regions, particularly Southeast Asia and Sub-Saharan Africa, experience the highest losses because of inadequate storage infrastructure, fragmented supply chains, insufficient transportation systems, and limited access to cold-chain technologies, with losses frequently exceeding 30–40% [4,24]. In contrast, developed regions such as North America, Europe, and East Asia have substantially reduced losses during production, storage, and distribution through mechanised harvesting, integrated cold-chain logistics, and advanced packaging systems. However, food waste remains significant at the retail and consumer levels owing to strict cosmetic standards, consumer purchasing behaviour, and market regulations that result in the rejection of edible fruits [28].

The consequences of postharvest losses extend beyond reduced marketable yield to include economic, nutritional, and environmental impacts. Fresh fruits are major sources of vitamins, minerals, dietary fibre, and bioactive compounds, yet their nutritional quality progressively declines during storage and distribution because of prolonged transportation and inappropriate handling [29]. These losses reduce farmer income, particularly among smallholder producers, while contributing to food insecurity, hunger, and inefficient use of land, water, and energy resources [30,31,32]. Consequently, improving postharvest management through affordable, effective, and sustainable preservation technologies has become a global priority to reduce food losses, strengthen food security, and enhance agricultural sustainability [33,34].

## 4. Biological Basis of Fruit Perishability

Fruit perishability is an intrinsic characteristic of harvested fresh produce and refers to the continuous decline in quality that occurs after harvest due to ongoing physiological and biochemical processes. The high perishability of fresh fruits primarily arises from their unique biological characteristics. Following harvest, fruits rely exclusively on their endogenous reserves of carbohydrates, organic acids, and other metabolites to sustain cellular maintenance and metabolic activity. As these reserves are progressively depleted, cellular homeostasis declines, structural integrity is compromised, and physiological deterioration gradually becomes irreversible [35]. Therefore, understanding these biological mechanisms provides the scientific basis for developing effective postharvest preservation technologies [15,16].

Among these physiological processes, respiration rate is one of the primary determinants because it regulates metabolic activity and the utilisation of stored substrates required for cellular function. Climacteric fruits exhibit a sharp increase in respiration and ethylene production during ripening, whereas non-climacteric fruits do not exhibit this respiratory peak [4,36]. Fruits with higher respiration rates generally exhibit faster ripening and shorter postharvest shelf life [15,37]. The underlying kinetics of this respiratory process are temperature-dependent, where higher storage temperatures immediately accelerate metabolic activity and hasten cellular senescence, whereas inadequate cold-chain compliance during transport and warehousing operations severely increases postharvest volume losses [38]. Thermal approaches, such as hot water treatment, can moderate respiratory activity through heat-induced physiological acclimation [5,13,24], whereas non-thermal technologies, including modified atmosphere packaging, controlled atmosphere storage, and edible coatings, regulate oxygen and carbon dioxide exchange to suppress respiration [37]. By slowing respiratory metabolism, these technologies delay quality deterioration, preserve cellular integrity, and extend postharvest shelf life [39]. Therefore, understanding respiratory metabolism provides an important physiological basis for selecting appropriate preservation strategies to maintain fruit quality and prolong storage life.

Similarly, ethylene sensitivity influences the shelf life of postharvest fruits [40,41]. As the primary ripening hormone, ethylene coordinates several physiological changes, including fruit softening, chlorophyll degradation, pigment development, aroma formation, and senescence [42]. While these processes are essential for normal fruit maturation, excessive or uncontrolled ethylene production accelerates quality deterioration and substantially shortens postharvest shelf life [36,42]. In contrast, non-climacteric fruits produce relatively low levels of ethylene and do not exhibit a climacteric respiratory burst. Their postharvest deterioration occurs primarily through transpiration, moisture loss, and gradual senescence rather than ethylene-driven ripening [13,15]. Ethylene is highly diffusible and can easily accumulate within storage rooms, transport containers, and retail environments. Consequently, ethylene produced by climacteric fruits may stimulate premature ripening of neighbouring ethylene-sensitive commodities, resulting in uneven fruit maturity, accelerated softening, increased susceptibility to microbial decay, and reduced market value. These effects are particularly significant in mixed-storage systems and long-distance export supply chains, where inadequate ventilation or poor atmospheric management can increase postharvest losses [30]. Thermal treatments, such as hot water treatment, can delay ripening by suppressing ethylene biosynthesis genes like 1-aminocyclopropane-1-carboxylate oxidase (ACO) and 1-aminocyclopropane-1-carboxylate synthase (ACS) [43]. Likewise, non-thermal technologies, including modified atmosphere packaging and controlled atmosphere storage, regulate the storage atmosphere or remove accumulated ethylene to reduce ripening rates and preserve fruit quality [44]. Collectively, these preservation strategies contribute to extending postharvest shelf life, reducing economic losses, and improving the sustainability of fresh fruit supply chains [45].

Another important determinant is the structural integrity of the fruit surface. The fruit cuticle serves as the primary protective barrier that regulates water loss while protecting internal tissues against mechanical injury, pathogen invasion, and environmental stress. Differences in cuticle thickness influence water loss, firmness retention, and susceptibility to postharvest decay. Consequently, fruits possessing thinner or structurally weaker cuticles are generally more susceptible to moisture loss, increased mechanical damage, and reduced storage potential compared with fruits having more robust cuticular structures [46]. To mitigate these limitations, both thermal and non-thermal postharvest interventions have been developed to preserve cuticle integrity and reduce surface deterioration. Mild thermal treatments, such as hot water treatment (HWT), can induce heat shock responses that stabilise cell membranes and reduce cuticular damage, therefore improving resistance to moisture loss and pathogen infection [47]. Similarly, non-thermal technologies, including edible coatings and modified atmosphere packaging, act as protective barriers that reduce transpiration, minimise mechanical injury, and maintain cuticle functionality, thereby preserving fruit firmness and extending storage life [40,41].

In general, tropical fruits are highly sensitive to cold storage temperatures [48], depending on the species, cultivar, maturity stage, and storage duration [49]. Storage below these commodity-specific temperatures induces chilling injury, one of the major physiological disorders limiting the postharvest storage of tropical fruits [23]. Unlike temperate fruits, tropical fruits are poorly adapted to prolonged exposure to low temperatures, making them highly susceptible to chilling damage. Consequently, chilling injury accelerates quality deterioration, shortens postharvest shelf life, and contributes significantly to postharvest losses throughout the supply chain [47]. Chilling injury disrupts normal physiological processes and is characterised by symptoms including surface pitting, peel discolouration, internal browning, water-soaked tissues, uneven or incomplete ripening, and increased susceptibility to microbial decay [41]. The severity of chilling injury varies among fruit species and cultivars. For example, bananas generally develop chilling injury below approximately 13–14 °C, whereas papaya, mango, and avocado are susceptible at temperatures below 10–13 °C, depending on the cultivar and stage of ripeness. These differences highlight the importance of establishing commodity-specific storage temperatures to minimise physiological damage while maintaining fruit quality during storage and transportation. Although refrigeration remains an effective approach for slowing respiration, ripening and senescence, it is insufficient as a stand-alone preservation strategy for many fruits, especially tropical fruits, because of their inherent chilling sensitivity [22,50]. Consequently, other postharvest technologies, including hot water treatment, intermittent warming, modified atmosphere storage, edible coatings, and antioxidant-based approaches, have been increasingly investigated to maintain fruit quality and extend postharvest shelf life [45]. For example, pre-storage HWT improves tolerance to subsequent cold storage by activating protective heat stress responses that stabilise cell membranes and reduce chilling injury, thereby helping to maintain fruit quality and extend postharvest shelf life [33,40]. These integrated preservation strategies are particularly important for supporting long-distance transportation and export markets, and reducing postharvest losses while preserving the commercial value of fresh fruits.

Enzymatic activity represents another major determinant of postharvest fruit deterioration and serves as a direct biochemical driver of many quality changes observed during storage. Following harvest, fruits remain metabolically active, and numerous endogenous enzymes continue to function, influencing texture, appearance, nutritional value, and storage performance. Among these, cell wall-modifying enzymes such as polygalacturonase (PG), pectin methylesterase (PME), cellulase, and β-galactosidase progressively degrade pectin and structural polysaccharides within the cell wall matrix, resulting in tissue softening, loss of firmness, and reduced resistance to mechanical damage [51,52]. Fruit softening is therefore not solely a consequence of ripening, but also an outcome of coordinated enzymatic disassembly of cell wall components. Similarly, oxidative enzymes including polyphenol oxidase (PPO) and peroxidase (POD) contribute significantly to enzymatic browning through the oxidation of phenolic compounds into dark-coloured pigments. These reactions negatively affect visual appearance and consumer acceptance while also accelerating quality deterioration under certain storage conditions. In addition, chlorophyll-degrading enzymes such as chlorophyllase contribute to colour changes associated with ripening and senescence, particularly during the transition from green to yellow or orange pigmentation. Recent studies highlight that fruit softening, browning, and colour degradation are strongly linked to these enzyme-mediated pathways, making enzymatic regulation an important target for postharvest preservation technologies [53,54]. Consequently, enzymatic mechanisms should be considered alongside respiration, ethylene metabolism, structural integrity, and chilling injury when evaluating the biological basis of fruit perishability.

However, these intrinsic biological characteristics do not function independently. Their influence on postharvest quality is continuously modified by external environmental conditions, including storage temperature, relative humidity, atmospheric composition, and mechanical handling [15,46]. These factors frequently accelerate internal metabolic processes by increasing respiration, water loss, oxidative stress, and pathogen susceptibility, thereby reducing shelf life if appropriate postharvest management practices are not implemented [55]. Understanding these interconnected mechanistic determinants provides the scientific basis for postharvest preservation technologies that underpin the selection of appropriate thermal and non-thermal postharvest interventions to maintain fruit quality and reduce postharvest losses. It is important to note that factors contributing to postharvest deterioration differ among fruit species because of variations in their physiology, ripening behaviour, and susceptibility to physical and microbial decay.

Microbial decay is one of the most prominent causes of postharvest fruit losses and should not be viewed solely as an external contamination issue. Although microorganisms such as fungi and bacteria originate from the surrounding environment, successful infection and disease progression are closely linked to host physiology. Recent studies have shown that the strong interaction between ripening progression and pathogen susceptibility is a key determinant of postharvest disease development and subsequent quality loss [56,57]. During ripening and senescence, fruits undergo structural, biochemical, and metabolic changes that weaken natural defence systems and increase susceptibility to pathogen invasion [56,58]. Progressive cell wall degradation, tissue softening, membrane destabilisation, moisture accumulation, and reductions in constitutive antifungal compounds collectively create conditions that favour pathogen establishment and colonisation [58]. Furthermore, microbial infection itself accelerates deterioration by producing cell wall-degrading enzymes, toxins, and metabolites that exacerbate tissue breakdown, increase respiration, stimulate senescence, and reduce market quality [59]. Therefore, microbial decay should be considered a dynamic deterioration pathway that both influences and is influenced by extrinsic and intrinsic physiological ageing processes.

**Figure 1:**
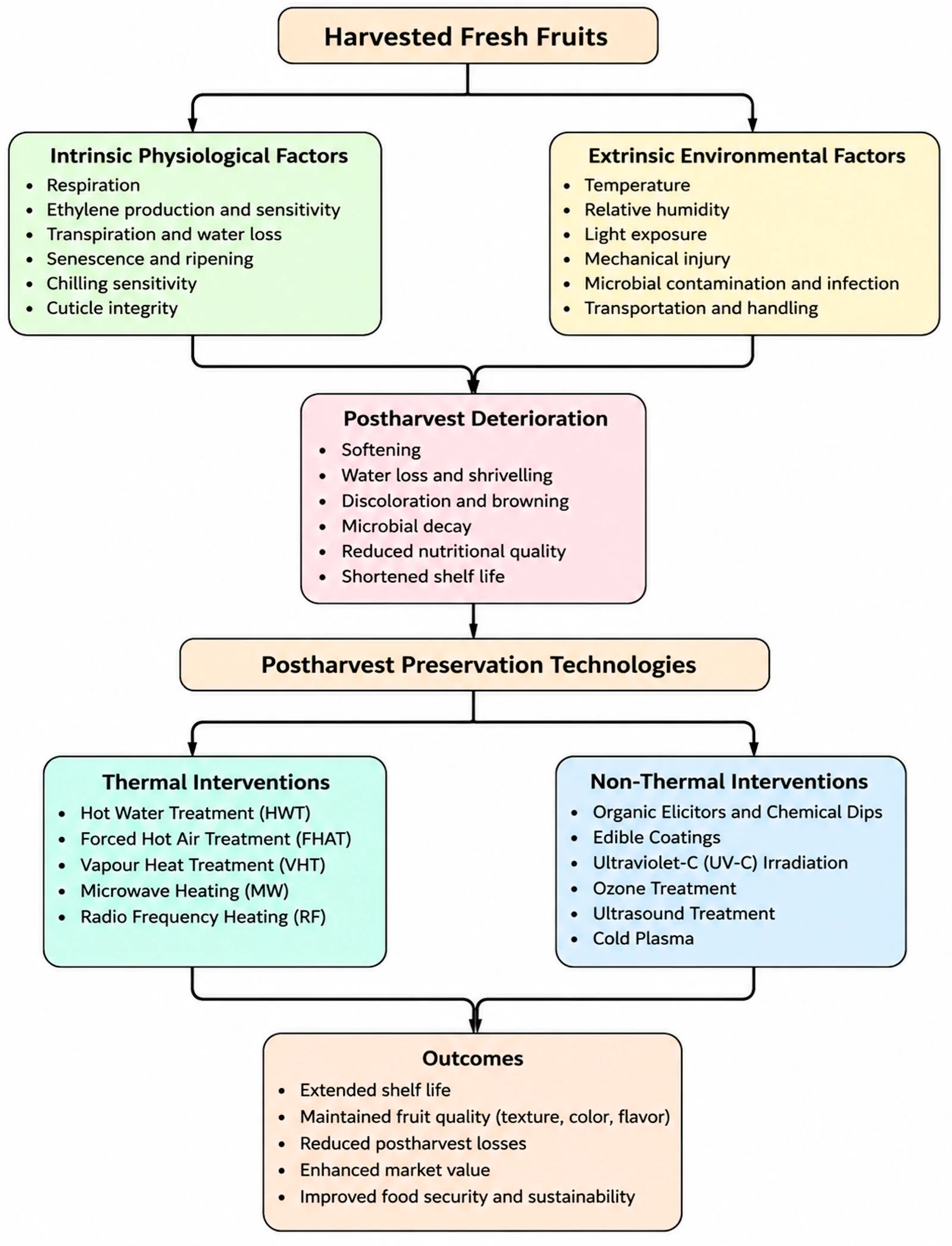
Conceptual framework illustrating the relationships among intrinsic physiological factors, extrinsic environmental factors, postharvest deterioration, and preservation technologies, and their outcomes in reducing postharvest losses and maintaining fruit quality.

Table 1 thus summarises the principal postharvest challenges affecting mango (*Mangifera indica*), banana (*Musa* spp.), and papaya (*Carica papaya*), which represent the most economically important tropical fruits worldwide [6]. Figure 1 presents a conceptual framework linking these physiological and environmental factors of postharvest fruit deterioration with preservation interventions.

Building on this conceptual map, the subsequent sections develop a comparative evaluation framework which translates these pathways and interventions into five implementation-oriented dimensions used throughout the review, as discussed below.

## 5. Comparative Evaluation Framework for Postharvest Preservation Technologies: Thermal and Non-Thermal

Thermal and non-thermal postharvest preservation technologies remain among the most established approaches for reducing PHL because of their long-proven efficacy in delaying ripening, suppressing microbial growth, and meeting phytosanitary requirements for domestic and international markets [29,55]. However, selecting an appropriate intervention technology extends beyond its ability to preserve fruit quality. Technologies demonstrating acceptable preservation performance under laboratory conditions may not necessarily be suitable for commercial translation because their adoption potential is influenced by cost, infrastructure availability, operational complexity, production scale, intended market, and regulatory requirements [29,42].

Existing reviews predominantly evaluate postharvest preservation technologies according to their biological performance, such as shelf life extension, microbial inhibition, maintenance of physicochemical quality, and nutrition retention [17,18]. While these parameters remain fundamental indicators of treatment effectiveness, they provide limited guidance for stakeholders responsible for technology selection in commercial supply chains, where financial, logistical, and operational considerations frequently determine implementation feasibility. To facilitate a more comprehensive evaluation, our review adopts an integrated evaluation framework to systematically compare thermal and non-thermal postharvest preservation technologies across five important implementation dimensions: (i) preservation efficacy, (ii) economic affordability, (iii) operational practicality, (iv) commercial scalability, and (v) production suitability. Collectively, these dimensions provide a stronger foundation for comparing technologies based on their biological efficacy and their potential for successful deployment across various postharvest contexts.

The chosen five-dimensional framework was operationalised as a structured, semi-quantitative evidence-synthesis approach rather than as a formal predictive or optimisation model. The framework was informed by established multi-criteria decision analysis (MCDA) approaches, commonly adopted for postharvest technology selection, where the technology alternatives are assessed against multiple technical, economic, and adoption-related criteria [66,67,68]. For each dimension, the assessment was based on predefined qualitative indicators extracted from the reviewed literature. These criteria were selected to reflect the technology-selection considerations identified in previous postharvest MCDA studies [66,67]. Ratings of Low, Moderate, and High were assigned in accordance to the direction and consistency of evidence across these indicators rather than by applying a universal numerical performance threshold. This is because the reviewed technologies are evaluated in varied experimental systems using different outcome measures, fruit commodities, treatment conditions, and reporting units; therefore, a common percentage-improvement threshold would not be directly comparable across technologies. Thus, the ratings represent an evidence-informed synthesis rather than a measurement of absolute performance. Consequently, the five dimensions were not given a universal numerical weighting because the relative importance of each criterion is context-dependent. For instance, affordability and operational simplicity may be prioritised in smallholder systems, whereas phytosanitary performance and scalability may carry greater importance in export-oriented packinghouses. Accordingly, Table 2 and Table 3, presented in Section 6 and Section 7, respectively, are to be interpreted as multidimensional technology profiles rather than as a single ranked score, to prevent implying that one technology is universally superior and instead to identify the trade-offs among efficacy, affordability, operational requirements, scalability, and production context.

### 5.1. Preservation Efficacy

One of the primary evaluation criteria of postharvest interventions involves an assessment of their efficacy in reducing quantity and quality losses [69]. Effective preservation technologies should be able to minimise physiological and biochemical changes, especially towards fruits undergoing ripening and senescence, suppress microbial infection, preserve nutritional composition, and maintain sensory attributes [49]. The biological efficacy of postharvest preservation technologies depends on their ability to modulate the physiological mechanisms responsible for postharvest deterioration. Thermal preservation methods rely on heat-induced inactivation to significantly lower microbial populations and can trigger defensive cellular mechanisms like the expression of heat shock proteins and elevated antioxidant activity [43]. Furthermore, elevated temperatures have been shown to reduce the activity of enzymes such as polyphenol oxidase (PPO), peroxidase (POD), polygalacturonase (PG), and pectin methylesterase (PME), which are involved in enzymatic browning, cell wall degradation, firmness loss, and senescence [70,71,72]. Consequently, enzyme inactivation represents an important mechanism through which thermal treatments preserve colour, texture, and overall fruit quality during storage and distribution. In contrast, non-thermal technologies protect fruit quality and extend shelf life at or near ambient temperatures [73,74]. This is achieved by modulating microbial activity, gas exchange, and oxidative pathways, without exposing the food matrix to degrading heat levels.

### 5.2. Economic Affordability

The adoption of postharvest technologies is significantly influenced by economic affordability, especially within developing nations due to restricted financial resources and infrastructure [17]. Even when preservation systems deliver promising biological efficacy, their commercial integration remains constrained if procurement, installation, and operational expenses surpass the financial capabilities of producers. Therefore, evaluations of preservation technology must balance its biological efficacy with an assessment of practical affordability. In general, systems requiring low initial and ongoing investments are ideally suited for decentralised networks and smallholder farmers [72]. Conversely, options demanding specialised facilities, continuous energy supplies, or specialised personnel are generally viable only for export operations and commercial packinghouses [75].

In this review, economic feasibility is assessed through a structured capital expenditure (CAPEX: equipment, installation, basic infrastructure) and operational expenditure (OPEX: energy, labour, maintenance, water, process, chemicals, and coating materials) lens following common techno-economic assessment practice in food-processing systems [76]. Accordingly, we adopted a semi-quantitative, benchmark-based approach to evaluate economic affordability. Quantitative cost values are reported only where sufficient data is available, while qualitative classifications are used where such economic data is limited. For available quantitative economic data, costs were converted to US dollars (USD) where appropriate. Instead of applying universal monetary thresholds across fundamentally different technologies, we evaluated thermal and non-thermal treatment methods individually against selected reference baseline methods from one of our chosen treatment methods. For thermal treatment, we discuss hot water treatment (HWT), forced hot air treatment (FHAT), vapour heat treatment (VHT), microwave heating, and radio frequency (RF) heating, where HWT served as the reference baseline. For non-thermal methods, we discuss organic elicitors and chemical dips, edible coatings, UV-C irradiation, ozone treatment, ultrasound, and cold plasma, with organic elicitors and chemical dips as the reference baseline.

For consistency, technologies were assigned to four relative affordability categories: Low, Moderate, High, and Very high. A Low classification indicates comparatively limited capital and operating requirements, minimal specialised infrastructure, and simple implementation. Moderate indicates additional dedicated equipment or moderate resource and technical requirements. High indicates substantial capital investment, energy, infrastructure, or specialised technical requirements. Very high indicates highly specialised equipment, substantial infrastructure requirements, and/or high technical expertise relative to the selected baseline reference method. These classifications were based on evidence from reported economic data, equipment requirements, process complexity, and implementation conditions, rather than on preservation efficacy alone. Where direct numerical cost information was unavailable, classifications were assigned from the reported technical and infrastructure requirements and were treated as evidence-informed comparative judgements. These assessments were then summarised into comparative affordability classes in Table 2 and Table 3.

For thermal treatments, hot-water treatment (HWT) was chosen as a quantitative reference baseline for economic affordability. HWT is widely recognised as a simple, environmentally safe, and commercially mature technology that has been implemented from smallholder supply chains to industrial export systems [77]. A study on a decentralised HWT plant for banana reported a capital investment of about 100,000 Bangladeshi Taka (≈US $1300) and a treatment cost of ~0.55 Taka kg^−1^ (≈US $0.007 kg^−1^, i.e., ≈US $7 per ton, t^−1^) in a lower-middle-income context [78]. These CAPEX and OPEX indicators show that HWT can operate with modest upfront investment and low treatment cost, and were therefore used as the baseline for interpreting affordability classes in this review. Benchmarking alternative thermal interventions against HWT showed steep increases in CAPEX and OPEX. For example, a study on fruit-fly disinfestation in mango showed that the cost of HWT is approximately 10% of the cost of VHT, reflecting a roughly ten-fold premium [79]. This difference is attributed to the need for high energy inputs for the system and more specialised equipment. On this basis, the affordability classes (“Low”, “Moderate”, “High”, “Very high”) reported in Table 2 and Table 3 are not purely qualitative judgements but are anchored to the empirically quantified HWT baseline and to documented comparative evidence where available. For non-thermal postharvest treatments, organic elicitors and chemical dips were chosen as the reference baseline. This is because they generally require only simple tanks or spray systems and rely on low-priced commodity chemicals, with minimal expenditure cost for chemical replenishment and water use. For instance, chlorine-based fungicide or sanitiser dips are still a go-to postharvest treatment method because they are technically effective and highly cost-effective, making them candidates for cost benchmarking when newer technologies such as ozonated water are evaluated [80].

Importantly, the affordability classifications presented in this review are intended as practical comparative guidance rather than universal economic rankings. Furthermore, our analysis is restricted to the technologies selected for this review and does not represent an exhaustive assessment of all available postharvest technologies. The same technology may also exhibit substantially different economic profiles depending on fruit commodity, treatment scale, equipment configuration, market energy prices, labour costs, infrastructure availability, geographic region, and intended market. Therefore, this framework should be interpreted as a decision-support guide for identifying relative economic burdens and technology-selection considerations, rather than as a substitute for specific techno-economic analysis.

### 5.3. Operational Practicality

Operational practicality refers to the technical viability of integrating selected postharvest technology into standard postharvest workflows. Although many technologies show encouraging outcomes in regulated laboratory settings, their viability for commercial use hinges on consistent execution, minimal procedural intricacy, and manageable resource allocations [81,82]. Postharvest technologies risk failing the transition from labs when they require overly precise operational execution that real-world packinghouse labour cannot replicate consistently [26]. Interventions that necessitate distinct engineering skills, prolonged system fine-tuning, or advanced tracking systems can encounter substantial hurdles to integration as the complex operational practicality quickly eclipses the technology’s theoretical efficiency gains. On the other hand, approaches utilising straightforward tools and requiring low worker instruction can be seamlessly incorporated into active postharvest routines, notably within resource-constrained environments [26]. As a result, assessing operational practicality offers crucial details on the translational viability of selected postharvest preservation technologies that go beyond laboratory settings.

### 5.4. Commercial Scalability

Many postharvest preservation technologies demonstrate promising experimental performance, but widespread commercial adoption remains scarce because of limitations associated with scale-up [73]. Commercial scalability therefore evaluates the capacity of a technology to maintain lab-proven preservation performance when transferred to industrial production systems. Scalable technologies should accommodate large processing volumes while maintaining treatment uniformity, operational efficiency, product consistency, and compliance with commercial food safety regulations [30,52]. Consequently, postharvest preservation technologies that seamlessly integrate into existing supply chain operations are generally more attractive to industry because they minimise the need for infrastructure overhauls while improving operational efficiency. Therefore, evaluating commercial scalability provides an important measure of whether a preservation technology can meet the increasing demands of modern fruit supply chains while supporting efficient commercial production and distribution [11,28]. Accordingly, commercial scalability in this review refers to the practicality of integrating preservation technologies into real-world supply chains.

### 5.5. Production Suitability

Due to the diverse biological traits of different fruits and the varying economic and infrastructural conditions, a single postharvest preservation technology cannot be universally implemented across all commodities or production models. Consequently, choosing the right technology requires a careful evaluation of the target production environment rather than focusing solely on the postharvest preservation technology’s biological efficacy. High-cost interventions that demand exact process control are often viable for large-scale, export-driven commercial operations managing premium fruits, yet they are typically infeasible for smallholder agricultural systems operating within local supply chains [66]. Conversely, inexpensive methods requiring minimal infrastructure setup can be more impactful, even if their preservation capabilities are relatively moderate. Evaluating production suitability therefore provides a practical assessment of technology by ensuring that preservation strategies are matched to the operational capacity and market objectives of different stakeholders throughout the postharvest supply chain [11,83].

## 6. Thermal Postharvest Technologies: Comparative Evaluation Based on Preservation Efficacy, Economic Affordability, Operational Complexity, Commercial Scalability, and Suitability for Different Production Systems

### 6.1. Hot Water Treatment (HWT)

Hot water treatment (HWT) is one of the most established and widely adopted thermal postharvest technologies because it combines effective disease control with low implementation costs. The preservation mechanism of HWT is based on immersing harvested fruits in water maintained at a controlled temperature for a predetermined period, allowing heat to be transferred by conduction from the surrounding water into the fruit tissues. This controlled heat exposure directly inactivates surface fungal pathogens and insect pests while simultaneously inducing beneficial physiological responses within the fruit. The preservation efficacy of HWT has been demonstrated in numerous tropical fruits. Treatment at approximately 45–55 °C for 3–10 min effectively suppresses *Colletotrichum gloeosporioides*, thereby reducing anthracnose incidence in mango (*Mangifera indica*) and papaya (*Carica papaya*), while immersion of banana (*Musa acuminata*) at 50–55 °C suppresses *Lasiodiplodia theobromae*, the principal causal agent of crown rot [33,45]. In addition to disease suppression, HWT preserves firmness and overall fruit quality through physiological adaptation to moderate heat stress, making it suitable for both domestic storage and export supply chains. Moderate heat stress stimulates the production of heat shock proteins (HSPs), enhances antioxidant defence systems, and stabilises cellular membranes, thereby delaying respiration, reducing chilling injury and slowing tissue softening during storage [7,65,84]. Consequently, HWT not only suppresses postharvest diseases but also improves fruit tolerance to storage-related stresses, contributing to prolonged shelf life without relying on synthetic chemical treatments. HWT may also reduce the activity of enzymes associated with enzymatic browning and tissue deterioration, particularly PPO and POD [70]. Furthermore, HWT was shown to disturb cell wall-degrading enzyme activity that contributes to delayed ripening, fruit softening, and loss of structural integrity [72]. Through these effects, HWT assists in preserving fruit colour, maintaining firmness, and slowing physiological deterioration during storage [85,86].

Among the thermal technologies evaluated in this review, HWT represents the most economically affordable option because the treatment system requires only basic equipment, including stainless-steel water tanks, heating elements, water circulation pumps, and temperature monitoring devices. Capital investment (CAPEX) and operating costs (OPEX) remain relatively low, as the process primarily consumes electricity or fuel for water heating and does not require specialised equipment or chemical inputs [65,84]. Operationally, HWT is straightforward to implement because treatment temperatures and immersion durations can be easily standardised with minimal technical expertise, making routine operation suitable for both research laboratories and commercial facilities.

From a commercial perspective, HWT is highly scalable because treatment systems can be adapted from small-batch units used by smallholder farmers to large continuous-processing systems operating in commercial packinghouses. Its low investment cost, ease of operation, and absence of chemical residues make it particularly suitable for resource-limited production systems while remaining compatible with commercial phytosanitary programmes for export markets [84]. Despite these advantages, HWT is not universally applicable. Treatment efficacy depends on precise optimisation of temperature and exposure time, as excessive heating may cause peel injury, uneven ripening, or internal tissue damage, whereas insufficient heating may fail to eliminate pathogens or insect pests [65].

Furthermore, responses vary among fruit species, cultivars, and maturity stages, requiring commodity-specific treatment protocols to maximise preservation efficacy while maintaining market quality. Although HWT remains the most practical and cost-effective thermal technology for many tropical fruits, its successful implementation ultimately depends on balancing treatment intensity with fruit physiological tolerance.

### 6.2. Forced Hot Air Treatment (FHAT)

Forced hot air treatment (FHAT) is a commercially established thermal postharvest technology that preserves fruit quality by exposing harvested fruits to a continuous flow of heated air under controlled temperature and humidity conditions. Unlike hot water treatment (HWT), FHAT transfers heat through forced air convection, allowing gradual and uniform heat penetration into fruit tissues without direct water contact. This heat exposure suppresses postharvest pathogens and insect pests while inducing beneficial physiological responses, including the activation of heat shock proteins and antioxidant defence systems, which delay ripening, reduce oxidative stress, and help maintain fruit quality during storage [12,82,87,88]. The technology has demonstrated good preservation efficacy in papaya (*Carica papaya*), persimmon (*Diospyros kaki*), and citrus fruits by reducing disease incidence while maintaining fruit firmness, colour, and overall quality during storage [89]. FHAT also contributes to quality preservation through heat-induced suppression of enzymes associated with browning and texture deterioration. Hot air exposure has been reported to reduce enzymatic activity involved in cell wall disassembly and oxidative deterioration, thereby helping maintain firmness, colour stability, and overall fruit quality during storage [52,86,90].

Compared with HWT, FHAT requires a relatively higher capital investment because it relies on insulated treatment chambers, heating units, and automated airflow systems with precise temperature control. Consequently, operating costs are moderately higher due to increased energy consumption. Nevertheless, the absence of water immersion eliminates post-treatment drying and reduces water consumption, making FHAT a practical alternative for regions with limited water resources [85].

Although FHAT provides more uniform heating than HWT and is readily scalable for commercial packinghouses and export facilities, its longer treatment duration, higher equipment costs, and greater operational complexity limit its suitability for smallholder production systems. Furthermore, treatment conditions must be carefully optimised, as excessive heat exposure may cause physiological injury, whereas insufficient heating may reduce treatment efficacy. Overall, FHAT offers an effective balance between preservation performance and commercial applicability, particularly for medium-to-large-scale postharvest operations.

### 6.3. Vapour Heat Treatment (VHT)

Vapour heat treatment (VHT) is a commercially established thermal technology primarily used as a phytosanitary treatment for fresh fruit exports. The preservation mechanism involves exposing fruits to saturated heated water vapour under controlled temperature and high-humidity conditions until the required core temperature is achieved. Heat is transferred gradually through moist air convection, allowing uniform heat penetration while minimising surface dehydration. This process effectively eliminates internal insect pests, particularly fruit flies, suppresses postharvest pathogens, and delays physiological deterioration without compromising fruit quality when treatment conditions are properly optimised [26,76,80].

VHT provides excellent preservation efficacy and is widely recognised as an approved quarantine treatment for export commodities such as mango (*Mangifera indica*) and papaya (*Carica papaya*), enabling compliance with international phytosanitary regulations while maintaining fruit quality [91]. Beyond quarantine disinfestation, VHT may contribute to quality retention through modulation of enzymes associated with oxidative browning and tissue degradation. For example, in fresh-cut pomegranate arils, short vapour-heat exposures (7–10 s) enhanced PAL activity while limiting the storage-related increases in PPO and POD, leading to better preservation of bioactive compounds and colour during storage [92]. These findings support the view that the controlled transfer of thermal energy into fruit tissues can slow enzyme-mediated quality loss while maintaining acceptable sensory and market characteristics when treatment conditions are appropriately optimised [86,90,92].

Despite its advantages, the effectiveness of VHT depends on precise control of treatment temperature, humidity, and exposure duration. Variations in fruit size, maturity, and cultivar can influence heat penetration, while excessive heating may result in physiological disorders such as internal tissue damage or uneven ripening. Compared with HWT and FHAT, VHT requires higher capital investment due to specialised treatment chambers, steam generation systems, and continuous core temperature monitoring. Operating costs are also higher because of the energy required to maintain saturated vapour conditions. Although the technology is highly practical for commercial packinghouses with established infrastructure and offers excellent scalability for export operations, its high installation cost and technical requirements limit its suitability for smallholder production systems. Nevertheless, VHT remains one of the most effective thermal technologies for quarantine disinfestation and export-oriented postharvest management, despite its widespread adoption being largely restricted to commercial operations where the higher investment is economically justified.

### 6.4. Microwave (MW) Heating

Microwave (MW) heating is an emerging thermal postharvest technology that preserves fruit quality through dielectric heating, whereby electromagnetic waves generate heat directly within fruit tissues by causing rapid oscillation of polar molecules, primarily water. Unlike conventional heating methods that rely on surface heat transfer, microwave energy produces rapid volumetric heating, resulting in efficient microbial inactivation and reduced enzymatic activity while minimising processing time [93]. The technology has shown promising preservation efficacy in fresh-cut fruits, thin-skinned fruits, and berries by reducing microbial contamination and inactivating PPO and POD, thereby maintaining colour, texture, flavour, and nutritional quality during storage [94]. Similarly, MW processing of acai berry markedly reduced POD and PPO activities, where the MWs could have disrupted hydrogen bonds and other weaker bonds, thus causing protein unfolding and structural changes that potentially inactivated the enzymes [95].

Despite its high preservation efficiency, MW heating requires substantial capital investment because specialised equipment, including industrial magnetrons, conveyor-based microwave systems, and electromagnetic shielding, is necessary for safe operation. Operating costs are also relatively high due to electricity consumption during microwave generation. Furthermore, the technology requires careful optimisation of microwave power, exposure time, and product loading, making its operation more technically demanding than conventional thermal treatments. Consequently, MW heating is currently more suitable for industrial food processing and commercial postharvest facilities than for smallholder production systems [17,96].

Although MW heating offers rapid processing and excellent preservation potential, uneven microwave energy distribution remains a major limitation. Variations in fruit size, shape, moisture content, and dielectric properties may produce localised hot and cold spots, leading to tissue damage or incomplete microbial inactivation. Therefore, further optimisation and equipment development are required before MW heating can be more widely adopted for commercial postharvest preservation of fresh fruits.

### 6.5. Radio Frequency (RF) Heating

Radio frequency (RF) heating is an advanced thermal technology that also utilises dielectric heating, but operates at lower frequencies than microwave systems, allowing deeper and more uniform heat penetration into larger fruit tissues. Electromagnetic fields generate heat throughout the commodity by inducing molecular movement within polar molecules, enabling rapid destruction of internal insect pests, fungal spores, and other quarantine organisms while preserving external fruit quality [75]. This uniform volumetric heating reduces the risk of surface overheating commonly associated with conventional thermal treatments and has shown promising results in kiwifruit, citrus, and other large fruits. RF heating in water was applied to vacuum-packed fresh-cut peaches at 70 and 80 °C for only 3–5 min and showed a marked reduction of up to ~85–94% of PPO activity, as compared to conventional heating, which required 30 min to reach similar inhibition levels, indicating that RF can markedly suppress browning-related enzymes with substantially shorter exposure times [97].

Among the thermal technologies evaluated in this review, RF heating requires the highest capital investment because the system incorporates high-power RF generators, customised electrodes, automated tuning systems, and electromagnetic shielding. Operating costs are similarly high due to substantial electricity consumption and the need for specialised maintenance and process calibration [75]. Consequently, RF heating is primarily implemented in industrial processing facilities and export packinghouses where high processing capacity and consistent treatment performance justify the investment. Its complexity and infrastructure requirements make the technology unsuitable for most smallholder farming systems.

Despite its advantages in heating uniformity and rapid pest disinfestation, successful RF treatment depends on careful optimisation of processing parameters and equipment design to achieve consistent energy distribution across different fruit sizes and cultivars. The high installation cost and technical expertise required continue to limit its commercial adoption. Nevertheless, RF heating represents one of the most promising next-generation thermal technologies for large-scale postharvest preservation and quarantine treatment as equipment design and process control continue to improve.

### 6.6. Comparative Synthesis of Thermal Technologies

The comparative evaluation demonstrates that no single thermal technology is universally superior, as each offers distinct advantages and limitations depending on the intended postharvest application and production scale [20,22]. Hot water treatment (HWT) remains the most economically affordable and operationally practical option, requiring minimal equipment and technical expertise while providing effective disease suppression and shelf life extension. These characteristics make HWT particularly suitable for smallholder farmers and decentralised postharvest facilities [26,36].

Forced hot air treatment (FHAT) offers improved heating uniformity and eliminates the need for water immersion, making it more suitable for commercial packinghouses where consistent processing and water conservation are important considerations, although this is accompanied by higher capital investment and energy requirements [40,98]. Vapour heat treatment (VHT) provides excellent preservation efficacy while serving as an internationally recognised phytosanitary treatment for export commodities, but its specialised infrastructure and operational costs largely restrict its application to export-oriented industries [5,99].

In contrast, microwave (MW) and radio frequency (RF) heating represent advanced thermal technologies that utilise dielectric heating to achieve rapid volumetric heat transfer and efficient microbial or insect pest control. These technologies offer significant advantages in processing speed, heating uniformity, and quality retention, particularly for high-value products. However, their widespread adoption remains constrained by high equipment costs, operational complexity, and the need for specialised technical expertise. Consequently, MW and RF heating are currently more applicable to industrial-scale processing than to smallholder production systems [65,87].

Overall, the selection of an appropriate thermal technology should be guided not only by preservation efficacy but also by economic affordability, operational practicality, commercial scalability, and suitability for different production systems. Balancing these factors is essential to maximising postharvest quality while ensuring that preservation technologies remain technically feasible and economically sustainable across diverse fruit supply chains. This comparative framework provides a practical basis for selecting thermal interventions according to specific production objectives and commercial requirements [75,84,93]. The comparative evaluation of these thermal postharvest technologies, based on preservation efficacy, economic affordability, operational practicality, commercial scalability, and production suitability, is summarised in Table 2.

**Table 2:** Comparative evaluation of major thermal postharvest technologies based on preservation efficacy, economic affordability, operational practicality, commercial scalability, and production suitability.

Technology	Preservation Efficacy	Economic Affordability	Operational Practicality	Commercial Scalability	Production Suitability	Reference
Hot Water Treatment	Effective against postharvest disease and extends shelf life	Relatively low treatment and investment cost	Simple operation with minimal technical expertise	Suitable for both small- and large-scale operations	Smallholder farms and rural facilities	[77,78]
Forced Hot Air Treatment	Heat is uniformly distributed and maintains fruit quality	Moderate level of operating cost	Requires controlled airflow and temperature	Highly suitable for commercial processing	Commercial export facilities	[100]
Vapour Heat Treatment	Very effective treatment and pest control	Moderately High operating cost due to specialised chambers and precise control systems	Needs specialised equipment and precise control of temperature	Widely adopted for export operations	Export industries and commercial packing houses	[100]
Microwave Heating	Rapid microbial inactivation and reduced enzymatic browning	High operating cost and high-energy- consumption-based equipment	Technical requirements expertise	High-value industrial processing facilities	Large-scale industrial processing and export industries	[19,97,98]
Radio Frequency Heating	Effective insect disinfestation	High investment and operating cost identified as major barrier to wider adoption	Needs complex operating systems with specialised expertise	Largely limited by cost; more suited for industrial applications	Large-scale industrial processing and export industries	[100,101,102,103,104]

## 7. Non-Thermal Postharvest Preservation Technologies: Comparative Evaluation Based on Preservation Efficacy, Economic Affordability, Operational Complexity, Commercial Scalability, and Suitability for Different Production Systems

Non-thermal postharvest preservation technologies extend fruit shelf life without exposing commodities to high temperatures, therefore minimising thermal damage while preserving nutritional value, sensory quality, and physicochemical integrity. These technologies contribute physical, chemical, or electromagnetic approaches to suppress microbial growth, delay physiological deterioration, and maintain postharvest quality through mechanisms other than heat [19,105]. Since each technology differs in preservation performance, implementation cost, and operational requirements, this section evaluates commonly applied non-thermal technologies based on the comparative evaluation framework.

### 7.1. Organic Elicitors and Chemical Dips

Organic elicitors and chemical dips preserve fruit quality by suppressing microbial growth, delaying ripening, and enhancing the natural defence responses of harvested fruits. Compounds such as salicylic acid (SA), hexanal, and 1-methylcyclopropene (1-MCP) regulate respiration, reduce ethylene action, and maintain antioxidant activity, and extend shelf life while preserving fruit firmness and nutritional quality [36,106]. These treatments exhibit high preservation efficacy, particularly in reducing postharvest diseases and delaying physiological deterioration. Economically, organic elicitors and chemical dips are among the most affordable non-thermal technologies because they require minimal investment and inexpensive treatment solutions. Operational complexity is low, as application only requires conventional spraying or dipping systems with limited technical expertise. These advantages provide good commercial scalability, enabling seamless integration into existing postharvest handling facilities. Consequently, this technology is highly suitable for both smallholder farms and commercial packinghouses, especially where cost-effective preservation methods are required. However, treatment effectiveness varies with fruit species, cultivar, and treatment concentration, requiring optimisation to maximise preservation performance while avoiding undesirable physiological responses.

### 7.2. Edible Coatings

Edible coatings preserve fruit quality by forming a semi-permeable barrier that regulates gas exchange, reduces moisture loss, and suppresses microbial growth. Coating materials such as chitosan, starch, and sodium alginate delay senescence, maintain firmness, and reduce postharvest water loss, thereby extending shelf life [40]. These coatings provide high preservation efficacy, particularly in maintaining fruit texture and reducing physiological deterioration. From an economic perspective, edible coatings are highly affordable because coating materials are inexpensive and biodegradable, and can easily be produced from renewable agricultural resources. Operational complexity remains low, requiring only simple dipping or spraying systems without specialised equipment. Their direct application supports commercial scalability, allowing implementation across diverse postharvest supply chains. Therefore, edible coatings are well suited for both smallholder producers and large commercial fruit packing facilities. Coating composition and thickness must be carefully optimised because excessive coatings may restrict gas exchange and negatively affect fruit quality.

### 7.3. Ultraviolet-C (UV-C) Irradiation

Ultraviolet-C (UV-C) irradiation preserves fruits by inactivating surface microorganisms while stimulating the fruits’ natural defence mechanisms. Controlled UV-C exposure enhances the accumulation of phenolic compounds and phytoalexins, improving resistance against microbial infection and delaying postharvest deterioration [64,107]. The technology demonstrates high preservation efficacy, particularly for controlling surface pathogens without leaving chemical residues.

Economically, UV-C requires moderate capital investment for irradiation equipment, although operating costs remain relatively low because only minimal electricity is required and no chemical inputs are needed [107,108]. Operational complexity is moderate, requiring accurate control of irradiation dose and exposure time. The technology exhibits high commercial scalability because it can be readily integrated into commercial packing lines and automated processing systems. Consequently, UV-C irradiation is most suitable for commercial packinghouses and export-oriented fruit industries, whereas adoption by smallholders may be limited by equipment costs. Excessive UV-C exposure may cause surface discolouration or quality deterioration, highlighting the importance of treatment optimisation.

### 7.4. Ozone Treatment

Ozone treatment preserves fruits through its strong oxidising activity, disrupting microbial cell membranes, damaging intracellular components, and reducing pathogen survival. Ozone also reduces respiration and modulates ethylene metabolism, thereby delaying fruit ripening and extending storage life [98]. This technology provides high preservation efficacy, particularly in suppressing microbial spoilage while leaving no chemical residues because ozone rapidly decomposes into oxygen. Economic affordability is moderate, as specialised ozone generators and monitoring systems increase initial capital investment, although operating costs remain relatively low because only electrical energy is required. Operational complexity is moderate, requiring continuous monitoring of ozone concentration to ensure treatment effectiveness and operator safety. The technology offers good commercial scalability, particularly in large storage facilities and commercial packinghouses. Therefore, ozone treatment is most suitable for commercial producers and export supply chains seeking residue-free preservation methods. However, excessive ozone concentrations may induce oxidative damage in sensitive fruit cultivars.

### 7.5. Ultrasound Treatment

Ultrasound treatment preserves fruit quality through acoustic cavitation, where microscopic bubbles rapidly form and collapse to generate mechanical forces capable of removing microbial biofilms and surface contaminants. This process reduces microbial populations while maintaining fruit texture, colour, and nutritional value [22]. Ultrasound demonstrates high preservation efficacy, particularly for fresh-cut fruits and minimally processed produce. The technology has moderate economic affordability because specialised ultrasonic equipment and higher energy consumption increase both capital and operating costs. Operational complexity is relatively high, requiring careful optimisation of ultrasonic frequency, treatment duration, and processing conditions. Despite these requirements, ultrasound provides moderate commercial scalability, particularly for industrial washing and fresh-cut fruit processing systems. Therefore, it is best suited for medium-to-large-scale industrial processors, while its adoption by smallholder producers remains limited. Excessive treatment intensity may damage delicate fruit tissues and reduce overall quality.

### 7.6. Cold Plasma

Cold plasma is an advanced non-thermal technology that generates reactive oxygen and nitrogen species, ultraviolet photons, and charged particles capable of inactivating microorganisms without significantly increasing fruit temperature. These reactive species disrupt microbial cell membranes and intracellular components, effectively reducing postharvest decay while preserving fruit texture, colour, and nutritional quality [55,109]. Among the reviewed technologies, cold plasma exhibits one of the highest preservation efficacies, particularly against microbial contamination. However, economic affordability is relatively low because specialised plasma generation systems and high-voltage power supplies require high capital investment. Operational complexity is high, as treatment parameters, gas composition, and exposure conditions must be carefully controlled to achieve preservation outcomes. Although cold plasma demonstrates strong commercial scalability for industrial processing and export industries, its widespread adoption is currently limited by equipment cost and technical requirements. Consequently, the technology is most suitable for high-value fruits and large commercial processing facilities rather than smallholder production systems. Furthermore, excessive treatment may damage fruit surface tissues, emphasising the need for careful optimisation.

### 7.7. Comparative Synthesis of Non-Thermal Technologies

The comparative evaluation demonstrates that non-thermal preservation technologies differ considerably in preservation efficacy, economic affordability, operational complexity, commercial scalability, and production suitability. Organic elicitors, chemical dips, and edible coatings remain the most affordable and operationally simple technologies, making them highly suitable for both smallholder farmers and commercial producers. UV-C irradiation and ozone treatment provide effective residue-free preservation with moderate implementation costs and excellent scalability for commercial packinghouses and export industries. In contrast, ultrasound and cold plasma offer superior microbial control and quality preservation but require greater capital investment, specialised equipment, and technical expertise, making them more appropriate for industrial processing facilities. Therefore, selecting an appropriate non-thermal technology should consider not only preservation efficacy but also economic feasibility, operational requirements, commercial scalability, and the intended production system. Table 3 summarises the comparative evaluation of major non-thermal postharvest technologies according to preservation efficacy, economic affordability, operational practicality, commercial scalability, and production suitability.

**Table 3:** Comparative evaluation of major non-thermal postharvest technologies based on preservation efficacy, economic affordability, operational practicality, commercial scalability, and production suitability.

Technology	Preservation Efficacy	Economics Affordability	Operational Practically	Commercial Scalability	Production Suitability	Reference
Organic Elicitors and Chemical Dips	High—Delays ripening, suppresses microbial growth, and enhances natural defense responses	Low implementation cost	Simple; minimal technical expertise	Readily incorporated into existing postharvest handling systems	Smallholder farms and postharvest handling facilities	[110]
Edible Coatings	High—Reduces moisture loss, delays senescence, and maintains firmness	Low implementation cost	Simple application system	Suitable for large-scale commercial systems	Commercial fruit industries and smallholder producers	[96,111]
UV-C Irradiation	High—Controls surface pathogens	Low-to-moderate operating cost	Requires specialised equipment	Readily suited for automated postharvest packing facilities	Postharvest handling facilities and export industries	[112]
Ozone Treatment	Highly effective microbial control	Moderate operating cost	Requires specialised ozone monitoring and safety control systems	Applicable to commercial storage and packing facilities	Export supply chain	[98,113]
Ultrasound	High—Reduces microbial contamination and maintains fruit quality	Moderate operating cost	Requires specialised ultrasonic equipment	Mainly suitable for industrial processing	Medium-to-large-scale industrial processors	[76,114,115]
Cold Plasma	Very high—Very good microbial inactivation and preserve quality	High investment cost	Requires skilled operators and specialised equipment	Commercial adoption is growing, yet it is restricted by high implementation costs	High value horticultural industries	[116,117]

## 8. Non-Destructive Quality Monitoring Technologies

As fruits undergo interventions such as hot water treatment, ozone exposure, and atmospheric cold plasma decontamination, the ability to monitor the internal quality without disrupting fruit integrity is critical for commercial postharvest operations. These technologies facilitate real-time monitoring of physical, physiological, and chemical changes across postharvest losses.

### 8.1. Monitoring Internal Fruit Chemistry

Near-infrared (NIR) spectroscopy is a non-destructive analytical technique that utilises electromagnetic radiation within the wavelength range of 780–2500 nm to characterise the internal chemical composition of fruits. The technique is based on the absorption of near-infrared light by the overtone and combination absorption of O-H, C-H and N-H molecular vibrations, which are abundant in water, sugars, organic acids, and other biochemical constituents [118]. Diffuse reflectance measurement is converted into absorbance using Equation (1) [119]:

(1) $$A=\log\left(\frac{1}{R}\right)$$

NIR spectroscopy generates spectral data that can be converted into quantitative measurements of internal fruit quality, particularly soluble solids content (SSC) and titratable acidity (TA), without destroying fruit integrity. This technique is particularly valuable for monitoring the effects of postharvest treatments such as hot water treatment (HWT); NIR enables assessment by detecting changes in sugar–acid balance and other internal physicochemical characteristics. Coupled with chemometric models such as partial least squares regression (PLSR), the technology provides real-time maturity predictions and automated quality classification to ensure product uniformity during storage and export [63].

### 8.2. Monitoring Structural Integrity

Hyperspectral imaging (HSI) integrates as one of the most advanced non-destructive technologies for postharvest fruit quality assessment because it can combine digital imaging with optical imaging and optical spectroscopy to obtain a measurement of fruit quality. HSI for food and agricultural applications are most commonly implemented in the visible–near-infrared (VNIR, approximately 400–1000 nm) and short-wave–near-infrared (NIR/SWIR, ~900–2500 nm) ranges for quality assessment of fruits and vegetables [120,121,122]. Beyond these ranges, thermal infrared HSI operating in the 8–14 µm (8000–14,000 nm) window has also been developed for agricultural monitoring, for example, for crop water-stress detection [123]. HSI generates a three-dimensional hyperspectral dataset containing both spatial and spectral information. This allows hidden bruises, internal defects, and early tissue damage to be detected before visible symptoms appear. After postharvest treatment such as ozone or atmospheric cold plasma, HSI can identify changes in tissue integrity in optical scattering and absorption associated with membrane disruption. Using PCA, the system can distinguish damaged tissue from healthy tissue during commercial packing [124,125].

### 8.3. Monitoring Metabolic Changes

Electronic noses (E-noses) integrate arrays of non-specific chemical sensors, most commonly metal oxide semiconductor (MOS) sensors, to detect volatile organic compounds (VOCs) released from fruits during storage. As VOCs interact with the heated sensor surface, typically at 200–400 °C, changes occur in the sensor’s electrical resistance relative to the baseline resistance due to the oxidation–reduction reactions (Equation (2)).

(2) $$S=\frac{R_{s}}{R_{0}}$$

where **R_s_** represents the resistance measured in the presence of VOCs, and **R_0_** is the baseline resistance in clean air [126]. The generated response patterns provide characteristic volatile fingerprints to monitor changes in fruit metabolism throughout storage. E-noses detect increases in fermentation-derived compounds such as ethanol and acetaldehyde, which accumulate under oxygen-limited conditions resulting from restricted gas exchange. Furthermore, these systems detect pathogen-associated VOCs produced by fungal pathogens, including *Colletotrichum* spp., allowing the early detection of disease, supporting rapid disease diagnosis and improving postharvest quality management [127].

### 8.4. Monitoring Technologies to Postharvest Treatments: Applications and Limitations

Within the five-dimensional framework presented in this review, non-destructive sensing tools are considered complementary components that assist in decision-making for specific classes of thermal and non-thermal treatments, rather than being standalone technologies [128]. NIR spectroscopy is primarily associated with monitoring internal quality changes (SSC, titratable acidity, internal disorders) during time-dependent storage; therefore, it is suited for treatments such as coating-based systems [129,130,131]. It can also be adopted to help verify that internal quality remains within acceptable specifications after thermal treatments such as HWT or cold plasma [132,133]. NIR systems are relatively mature and commercially available for in-line and portable measurements, but practical deployment depends on application-specific calibration and ongoing model maintenance across cultivars and seasons, and higher-end devices can still represent a substantial capital investment [118,134]. HSI, by contrast, can detect the physiological changes in the near-surface cellular structure immediately after a stress event occurs [135,136]. HSI has already been successfully integrated with several surface-oriented treatments, including UV-C surface treatments on bananas to detect skin damage and quality loss [137] and HWT followed by drying for apple slices to monitor changes in colour and moisture [138], as well as under cold storage and MAP to detect chilling injury and deterioration [135]. Consequently, HSI may be adopted for thermal treatments like HWT, FHAT, VHT, and others since they carry a significant risk of inducing thermal scald, skin browning, and near-surface tissue collapse if temperature/time thresholds are exceeded. While HSI provides rich spatial–spectral information and is highly sensitive to subtle surface defects, current systems remain relatively capital-intensive and computationally demanding, with high equipment cost, large data volumes, and complex analysis pipelines cited as key obstacles to routine industrial adoption [139]. E-noses are emerging as complementary monitoring tools suited across both thermal and non-thermal postharvest technologies since they primarily provide time-resolved fingerprints of volatile organic compounds (VOCs) associated with ripening, off-odours, and pathogen activity [140]. For instance, during cold storage (5 °C), an E-nose was able to detect fungal infections like *Botrytis cinerea* in strawberries within 24 to 48 h, long before visual decay symptoms appear [141]. Besides that, an E-nose was utilised to discriminate sweet cherries stored in air versus high-CO_2_ MAP during cold storage, in line with visual quality and antioxidant activity monitoring [142]. In contrast to NIR and HSI systems, E-noses are relatively low-cost, with portable sensor arrays that offer automated, high-throughput VOC monitoring, making per-measurement costs small compared with chromatographic analyses, although initial setup cost remains relatively high [143]. However, E-noses are highly sensitive to temperature and humidity and prone to cross-sensitivity, and require careful calibration and compensation models to ensure stability and transferability across commodities and storage environments, which highlights their operational complexity [140].

Overall, these “treatment + sensor” pairings are subject to important practical limitations. NIR systems are mature, but calibration models may be cultivar-, season- and instrument-specific, thus requiring careful standardisation and calibration when deployed across varied fruit supply chains [132]. HSI provides spatial information and is highly sensitive to subtle surface defects, yet camera and illumination units are considerably more capital-intensive, generate large data volumes, and require advanced chemometric or image-analysis pipelines, which can restrict large-scale adoption [144]. In contrast, E-nose systems offer low cost with real-time VOC monitoring, but can be easily affected by temperature, humidity, and background atmosphere. They also rely on pattern-recognition models and proper calibration to ensure specificity and transferability across fruit types and storage facilities [140]. In this review, these tools are therefore recommended not as universally required add-ons, but as context-dependent options that can be selectively combined with affordable preservation technologies to enhance quality assurance and reduce avoidable postharvest losses, particularly where export value, regulatory requirements, or risk of defects justify the added complexity and cost.

## 9. Comparative Perspectives on Thermal and Non-Thermal Technologies

The optimisation of the fresh fruit supply chain requires balancing the physiological and thermodynamic trade-offs between thermal and non-thermal technologies. Nevertheless, postharvest research evaluates these treatments under controlled laboratory conditions, failing to account for their interactions within integrated supply chain systems. An effective commercial strategy may benefit from carefully designed multi-modal hurdle approaches, where complementary low-energy interventions are combined to achieve required preservation outcomes while avoiding damage to postharvest produce. However, the number of studies that directly compare combined interventions with any corresponding single treatments remains limited, and true synergistic, additive, or antagonistic interactions have not yet been systematically quantified across technologies and fruit commodities. The major challenge in tropical fruit postharvest management is understanding the trade-offs between thermal and non-thermal preservation methods.

Although most published studies still evaluate preservation technologies in isolation, there is a growing body of work on combined or sequential interventions [110,145,146]. These studies generally report additive or modestly synergistic reductions in decay and quality loss relative to the corresponding single treatments but also highlight potential trade-offs such as increased tissue stress or higher operational complexity [146]. Combination hurdle treatments were shown to exhibit non-linear interactions, initially designed for synergistic preservation; undesirable antagonistic effects frequently occur when process parameters are not optimised for specific commodities [110,146]. Furthermore, combined treatments were also shown to concurrently induce synergistic benefits on structural parameters like firmness and ethylene suppression but also antagonistic trade-offs on bioactive retention and antioxidant capacity [145]. Establishing rigorous, multi-arm experimental protocols is therefore crucial to validate and optimise combined technologies to ensure they consistently deliver added preservation gains such that they justify any added operational cost [129].

Individually, among thermal technologies, HWT provides an effective, chemical-free solution for controlling anthracnose and quarantine pests. Nevertheless, its effectiveness depends on accurate temperature and exposure times, beyond which heat injury may occur [45]. At the biochemical level, moderate heat treatment regulates postharvest ripening by inhibiting ethylene biosynthesis and stimulating heat shock proteins, thereby enhancing membrane stability and reducing oxidative damage [41], including by catalase and superoxide dismutase, which can lead to heat injury, tissue breakdown, and accelerated fruit softening [9]. Unlike thermal treatments, non-thermal technologies such as ozone and cold plasma preserve heat-sensitive compounds, including bioactive molecules, ascorbic acid, and pigments, while providing antimicrobial activity by generating reactive oxygen and nitrogen species to react with the pathogens [147], but the long-term physiological effects on fruit tissues are still unclear [109].

The long-term physiological effects of reactive oxygen and nitrogen species on fruit tissues may damage the cuticle and cell membranes, resulting in increased water loss and faster quality deterioration. The adoption of these technologies is slightly constrained by economic and infrastructural limitations [49]. The commercial feasibility of postharvest technologies depends on regional economic and infrastructural capacity; while non-thermal technologies achieve superior quality retention, their high capital costs and infrastructure requirements limit their adoption in developing regions. As a result, thermal technologies continue to be the most practical and accessible preservation strategy for smallholder production systems [49].

Overall, the choice of postharvest preservation technologies should be guided by regional infrastructure and commercial needs. Future studies can also rigorously test hurdle strategies that combine complementary thermal and non-thermal approaches with real-time monitoring techniques. Based on the comparative profiles assembled in this review, such integrated strategies are hypothesised to improve preservation efficiency, maintain fruit quality, extend shelf life, enhance disease management, and reduce postharvest losses under diverse commercial conditions, but their net gains relative to optimised single interventions remain to be demonstrated [22,36,47].

The findings throughout this review state that no single preservation technology is suitable for all fruit commodities or production systems. Instead, preservation technology selection should be guided by the interaction between fruit physiology, the objectives of production, the availability of infrastructure, and economic feasibility. Consequently, preservation technologies should be chosen according to a decision-making framework rather than solely preservation efficacy. Building on the five-dimensional evaluation framework, Table 4 presents a guide for decision-making for selecting postharvest preservation technologies based on specific needs. However, a key limitation identified throughout this review is the relatively low number of combined thermal and non-thermal treatment studies that directly compare hurdle strategies with their constituent single technologies under realistic supply-chain conditions. Most of these studies focus on disease control and basic quality indices, with limited attention to operational complexity and cost [148,149,150,151]. As a result, while our integrated framework and the decision matrix in Table 4 are grounded in the best available evidence on individual technologies and selected treatment–monitoring pairings, they should be regarded as evidence-informed, testable design hypotheses. Several pairings are presented as conceptual combinations from our comparative evaluation of single technologies, existing combination treatments, and monitoring tools to guide future experimental design.

## 10. Future Horizons and Digital Ecosystems

The future of postharvest fruit preservation is expected to combine biological preservation technologies with digital innovations to improve fruit quality and reduce postharvest losses throughout the supply chain. Instead of depending on a single preservation method, future strategies are likely to integrate molecular and digital technologies to provide better disease control, maintain fruit quality, and support real-time decision-making during storage and transportation. Nanotechnology is becoming an important area in the development of next-generation edible coatings. Conventional edible coatings often have limitations such as limited structural stability, uneven coating thickness, and limited microbial activity. To overcome these problems, researchers have developed nano-based edible coatings using materials such as nano-chitosan and cellulose nanocrystals combined with natural antimicrobial compounds, including plant essential oils [42]. From an efficacy perspective, nano-enabled coatings provide strong barrier properties and improved pathogen control, and can also be used for partial ethylene scavenging [167,168]. These nano-bioformulations improve the stability of the coating and allow a more controlled release of microbial compounds, thus helping to reduce microbial growth while maintaining fruit quality during storage [169].

Another promising area is molecular biotechnology. Gene-editing technologies such as CRISPR-Cas9 have been investigated to regulate genes involved in ethylene biosynthesis, particularly ACS and ACO, which play crucial roles in fruit ripening [42]. In tomato, CRISPR/Cas9 has been used to modify key genes like FIS1 and PL to regulate firmness for enhanced storage and ethylene-related pathways, enabling delayed softening and extended shelf life while maintaining basic quality attributes [170,171]. In addition, researchers are also suggesting a way to enhance the natural antioxidant defence system by increasing the expression of enzymes such as catalase (CAT) and superoxide dismutase (SOD), which helps enhance the fruit’s ability to withstand oxidative stress and chilling injury during cold storage [87]. Within the framework of this review, gene-edited cultivars represent a potentially powerful tool for preservation efficacy, but their operational practicality and commercial scalability are currently limited by regulatory approval processes and consumer acceptance issues, especially in low- and middle-income markets; thus, they are best viewed as a long-term complement rather than an immediately deployable alternative to existing postharvest treatments [172,173].

Digital technologies are also expected to play an increasingly important role in future postharvest management. Smart sensors can continuously detect environmental conditions such as temperature, relative humidity, ethylene concentration, and carbon dioxide throughout storage and transportation [47]. The collected data can be analysed using cloud computing and artificial intelligence to estimate fruit quality and predict the remaining shelf life in real time. This information allows supply chain operators to make faster decisions, improve inventory management, and eliminate unnecessary postharvest losses. In terms of the dimensions in this review, simple data loggers and basic IoT (Internet of Things) sensors now offer high monitoring value and good economic affordability for many exporters and larger packhouses, whereas fully integrated IoT–cloud–AI (artificial intelligence) platforms that combine multi-sensor streams like temperature and gas composition with predictive models of fruit quality are still capital- and skill-intensive, and therefore more suited for high-throughput operations or high-value export chains [174]. AI and machine-learning models can serve decision-support roles that enhance existing thermal and non-thermal interventions. By combining sensor data, historical treatment records, and logistics information, AI models have been used to predict remaining shelf life, classify quality grades, and optimise cold-chain supply, which strengthens preservation efficacy and operational practicality of conventional technologies [174].

Overall, the future of postharvest preservation is moving towards integrated systems that combine advanced preservation technologies with digital monitoring tools. Combining nanotechnology, molecular biotechnology, and smart sensors is expected to improve fruit quality, extend shelf life, reduce postharvest losses, and support more sustainable fruit supply chains. Within the comparative framework used in this review, emerging nano-coatings and gene-edited cultivars primarily enhance preservation efficacy but currently face moderate-to-high economic and regulatory barriers, while smart sensors, IoT platforms, and AI models mainly strengthen monitoring and decision support, with affordability ranging from accessible (stand-alone loggers) to high (fully integrated sensor–cloud–AI ecosystems) [167,168,174]. Nevertheless, additional studies are still required to evaluate the commercial feasibility, economic cost, and large-scale implementation of these technologies under diverse production and storage conditions.

## 11. Conclusions

Postharvest loss (PHL) of fruits continues to be one of the major challenges affecting global food systems, especially as the world’s population grows and natural resources become increasingly limited. Fruits remain biologically active after harvest, and continued respiration, transpiration, ripening, and senescence, together with inappropriate handling, transportation, and storage conditions, accelerate quality deterioration and microbial spoilage. As a result, high postharvest losses reduce farmers’ income, disrupt market supply, threaten food and nutritional security, and contribute to unnecessary environmental impacts through the waste of land, water, energy, and other agricultural resources.

Reducing postharvest losses therefore represents a practical and sustainable way to improve food availability without the need to expand agricultural land or increase production intensity. A wide range of thermal and non-thermal preservation technologies has shown promising results in maintaining fruit quality and extending storage life. Nevertheless, many of these technologies require substantial financial investment, specialised equipment, better infrastructure, and technical expertise. In addition, regulatory requirements and consumer acceptance may limit their large-scale adoption, particularly among smallholder farmers in developing regions. Therefore, no single preservation technology is universally suitable, highlighting and the need for context-specific postharvest management strategies that integrate appropriate technologies, good handling and storage practices, supportive policies, and farmer education. Future efforts should therefore prioritise accessible and scalable postharvest solutions that balance preservation efficacy with economic feasibility and practical implementation, particularly for smallholder and resource-limited production systems.

## Figures and Tables

**Table 1:** Key postharvest challenges affecting mango (*Mangifera indica*), banana (*Musa* spp.), and *papaya* (*Carica papaya*) [60].

Fruit	PHL Classification	Core Factors	Symptoms	Reference
Mango (*Mangifera Indica*)	Respiration and ageing	High respiration rate and cell wall degradation	Flesh overripe, soft and watery	[61]
	Mechanical injury	Sap burn	Darkened skin and sap-burned blemishes	[62]
	Pathological attack	Latent infection of *Colletotrichum gloeosporioides*	Sunken lesions	[63]
Banana (*Musa* spp.)	Ethylene sensitivity	Autocatalytic ethylene production	Premature ripening	[35]
	Mechanical injury	Compression and impact damage	Skin blackening and rapid tissue softening	[34]
	Pathological attack	Fungal pathogen infection	Severe crown rot	[64]
Papaya (*Carica* *Papaya*)	Transpiration	High water Content	Severe surface damage skin browning	[15]
	Mechanical injury	Late leakage following physical injury	Tissue collapse and deformation	[65]
	Pathological attack	Skin damage increases fungal infection	Wet rot	[45]

**Table 4:** Decision-making framework for selecting appropriate postharvest preservation technologies. The table operationalises the five-dimensional framework by translating these dimensions into context-specific recommendations for thermal and non-thermal interventions across different fruit types and supply-chain situations, while indicating the current level of evidence for single-technology and combined-treatment approaches.

Decision Factor	Recommended Thermal Technology	Recommended Non-Thermal Technology	Evidence Status
Highly climacteric fruits (banana, mango, papaya, guava)	HWT, FHAT, VHT	1-MCP, ozone treatment, edible coating	- Single- and combined-treatment studies. - HWT and other heat-based treatments help reduce microbial contamination and maintain quality in fruits [20,77]. - Combination of HWT + 1-MCP showed improvement in “Solo” papayas relative to individual treatments [152]. - 1-MCP use in climacteric fruits like banana, mango, and papaya is based primarily on its single-use evidence, but studies have shown enhanced efficacy when 1-MCP is combined with other postharvest treatments to extend storage life [153]. - Ozone and edible coating (guar gum) have shown quality improvements in papaya, and their combined use improved shelf life and antioxidant content [154].
Thin-skinned fruits	Short-exposure HWT or FHAT	Organic elicitors and chemical dips, edible coatings, UV-C irradiation	- Combined treatment studies. - Mild heat and HWT are widely documented for decay control and quality preservation in soft fruits and vegetables [20]. - Edible coatings like chitosan improved shelf life and quality in table grapes [155]. - Edible coating + UV-C/ hot air/organic elicitors have been shown on various fruits where the combination better reduced decay and maintained phytochemicals [156]. - These findings allow us to recommend mild thermal (hot air/HWT together with organic elicitors or edible coatings and, where feasible, UV-C as evidence-based multi-hurdle options for thin-skinned, damage-prone sensitive fruits.
Export supply chain	HWT, VHT, microwave heating, RF heating	Cold plasma, ultrasound	- Single-technology studies and combined treatment studies. - VHT was shown as an effective quarantine intervention to disinfest oriental fruit flies in export papayas [157]. - RF energy provides a rapid, volumetric thermal treatment, whereas cold plasma serves as a non-thermal phytosanitary option for postharvest disinfestation in fresh fruits processing [103,116]. - Chosen technologies must be highly compliant on export regulatory requirements, which can drive up overall operational cost.
Smallholder farmers	HWT (simple tanks or basins)	Organic elicitors, chemical dips, edible coatings	- Single-technology studies - Studies on HWT alone showed disease reduction and prolonged shelf life using relatively simple equipment; edible coatings like chitosan were shown extend shelf life and reduce microbial growth in various fruits; 1-MCP + HWT also showed added net benefits [20,152,155,158]. - These supports recommending HWT and simple organic elicitors and chemical dips, edible coatings for smallholder and decentralised packing operations. - Adoption of postharvest technologies must be balanced with comparatively small margins.
Large commercial packinghouses	FHAT, microwave heating, RF heating	Cold plasma, ozone treatment, cold storage, edible coatings, ultrasound, MAP + edible coatings, ozone treatment + edible coating	- Single-technology studies and combined treatment studies; multi-hurdle integration mainly conceptual. - A recent review on RF heating summarised that RF systems are used for disinfestation of fresh fruits, emphasising its rapid and suitability for high-throughput systems with scale-up potential [159]. - Another study reviewed cold plasma systems for fruits, highlighting its rapid treatment, which makes it useful for commercial settings [160]. - Edible coatings and combined non-thermal hurdle treatments have shown that they can outperform single treatments [161]. - More room for hurdle treatments as larger industry players can invest more on research and development to optimise their preservation strategies.
Chilling-sensitive tropical fruits	HWT	Antioxidants, ozone treatment	- Single-technology studies and combined treatment studies; multi-hurdle integration mainly conceptual. - HWT has been shown to alleviate chilling injury in banana and zucchini fruits [162,163]. - Alginate and GABA coating reduced chilling injury in rose apples and mango respectively [164,165]. - Some studies combine pre-conditioning (e.g., HWT or hot air) with cold storage under modified atmospheres, but rigorously tested, standardised multi-hurdle protocols that integrate thermal pre-conditioning, intermittent warming, atmosphere control, and ozone for specific tropical fruit supply chains remain relatively scarce [166].

## Data Availability

No new data were created or analysed in this study. Data sharing is not applicable to this article.

## Abbreviations

The following abbreviations are used in this manuscript:

Abbreviation	Full Term
AI	Artificial Intelligence
ACS	1-Aminocyclopropane-1-Carboxylic Acid Synthase
ACO	1-Aminocyclopropane-1-Carboxylic Acid Oxidase
E-nose	Electronic Nose
FAO	Food and Agriculture Organisation of the United Nations
FHAT	Forced Hot Air Treatment
HSI	Hyperspectral Imaging
HWT	Hot Water Treatment
MAP	Modified Atmosphere Packaging
MW	Microwave Heating
NIR	Near-Infrared Spectroscopy
OECD	Organisation for Economic Co-operation and Development
PHL	Postharvest Loss
RF	Radio Frequency Heating
ROS	Reactive Oxygen Species
SA	Salicylic Acid
UV-C	Ultraviolet-C Irradiation
VHT	Vapour Heat Treatment
